# Supplementary material for: Asymptomatic gallstones: Cumulative incidence proportion, incidence rate, and risk factors for symptoms development: Systematic review and meta-analysis
Source: PLoS One. 2026 Mar 26;21(3):e0345462. doi: 10.1371/journal.pone.0345462 (PMC13020817; doi:10.1371/journal.pone.0345462)
Supplement: S1 Appendix — (DOCX) [file pone.0345462.s001.docx]

**Leave-one-out sensitivity analysis result.**

- **Risk Factors.**

For the male gender, leave-one-out sensitivity analysis confirmed the significance of the pooled effect, and heterogeneity ranged from 79.2% to 87.3% after excluding each study, S3-C Fig. For chronic liver disease, leave-one-out sensitivity analysis was not feasible due to the limited number of studies. Leave-one-out sensitivity analysis confirmed the non-significance of the pooled estimate for female gender, with heterogeneity ranging from 90.9% to 94.6%, **S8-A Fig** . For age, exclusion of Yousef Thwayeb (2004) showed that age <55 years was associated with an increased risk of developing symptomatic gallstones (MD = –8.04, 95% CI: –9.36 to –6.72, p < 0.0001), with heterogeneity reduced to I² = 85.7%, **S8-B Fig** . For smoking, the exclusion of Shabanzadeh, DM (2017) shifted the pooled estimate to a significant risk factor (RR = 1.10, 95% CI: 1.06–1.13, p < 0.0001) and reduced heterogeneity to I² = 0%, as shown in **S8-C Fig** . For the remaining risk factors, leave-one-out sensitivity analysis was not feasible due to the limited number of studies.

- **Complications.**

For complicated events, leave-one-out sensitivity analysis confirmed the non-significance of the pooled estimate, and heterogeneity ranged from 80.1% to 88.3% after excluding each study, **S9-A Fig** . Leave-one-out sensitivity analysis revealed consistent overall proportions and heterogeneity for adenocarcinoma of the gallbladder and obstructive jaundice after excluding each study, **S9-B and S9-C Fig** . For common bile duct stones, the overall proportion remained consistent, and heterogeneity dropped to I² = 0% after excluding Shabanzadeh, DM (2017) **S10-A Fig**. For biliary pain, the overall proportion ranged from 0.46 to 0.68, and heterogeneity ranged from 77.8% to 89.3% after excluding each study **S10-B Fig**. For acute cholecystitis, the overall proportion and heterogeneity changed to 0.14 (95% CI: 0.11–0.19; I² = 0%), **S10-C Fig**, and for gallstone pancreatitis, they changed to 0.04 (95% CI: 0.02–0.08; I² = 0%) after excluding Morris-Stiff (2023), **S10-D Fig**.

**Publication Bias result.**

For the male gender, Funnel plot inspection indicated asymmetry, with statistical evidence of publication bias (LFK = –5.92) **S4-C Fig**. Funnel plot inspection revealed asymmetry, with statistical evidence of publication bias for female gender, age, and smoking (LFK = –6.19, 4.21, and 2.5, respectively) **S11 Fig**. Funnel plot inspection for risk of presenting with a complicated event indicated an asymmetrical distribution of studies, with statistical evidence of publication bias (LFK = 4.47), **S12 Fig**. Funnel plot inspection indicated an asymmetrical distribution of studies, with statistical evidence of the presence of publication bias for biliary pain (LFK = 4.53), common bile duct stones (LFK = -1.65), adenocarcinoma of the gallbladder (LFK = 6.7), obstructive jaundice (LFK = -2.3), acute cholecystitis (LFK = -8.09), and gallstone pancreatitis (LFK = -8.13), **S13 and S14 Fig**.
