# Supplementary material for: Asymptomatic gallstones: Cumulative incidence proportion, incidence rate, and risk factors for symptoms development: Systematic review and meta-analysis
Source: PLoS One. 2026 Mar 26;21(3):e0345462. doi: 10.1371/journal.pone.0345462 (PMC13020817; doi:10.1371/journal.pone.0345462)
Supplement: S1 Table — (DOCX) [file pone.0345462.s003.docx]

| Electronic Database | Search Strategy |
| --- | --- |
| PubMed | (((((((asymptomatic gallstone*) OR (silent gallstone*)) OR (asymptomatic cholelithiasis)) OR (silent cholelithiasis)) OR (Non-complicated gallstone*)) OR (Incidental gallstone*)) OR (Non-complicated cholelithiasis) ) OR (Incidental cholelithiasis) |
| Scopus | (((((((asymptomatic gallstone*) OR (silent gallstone*)) OR (asymptomatic cholelithiasis)) OR (silent cholelithiasis)) OR (Non-complicated gallstone*)) OR (Incidental gallstone*)) OR (Non-complicated cholelithiasis) ) OR (Incidental cholelithiasis) |
| Web Of Science | (((((((ALL=(asymptomatic gallstone*)) OR ALL=(silent gallstone*)) OR ALL=(asymptomatic cholelithiasis)) OR ALL=(silent cholelithiasis)) OR ALL=(Non-complicated gallstone*)) OR ALL=(Incidental gallstone*)) OR ALL=(Non-complicated cholelithiasis)) OR ALL=(Incidental cholelithiasis) |
| Science Direct | (((((((asymptomatic gallstone) OR (silent gallstone)) OR (asymptomatic cholelithiasis)) OR (silent cholelithiasis)) OR (Non-complicated gallstone)) OR (Incidental gallstone*)) OR (Non-complicated cholelithiasis) ) OR (Incidental cholelithiasis) |
