# Supplementary figures and images for: Asymptomatic gallstones: Cumulative incidence proportion, incidence rate, and risk factors for symptoms development: Systematic review and meta-analysis
Source: PLoS One. 2026 Mar 26;21(3):e0345462. doi: 10.1371/journal.pone.0345462 (PMC13020817; doi:10.1371/journal.pone.0345462)

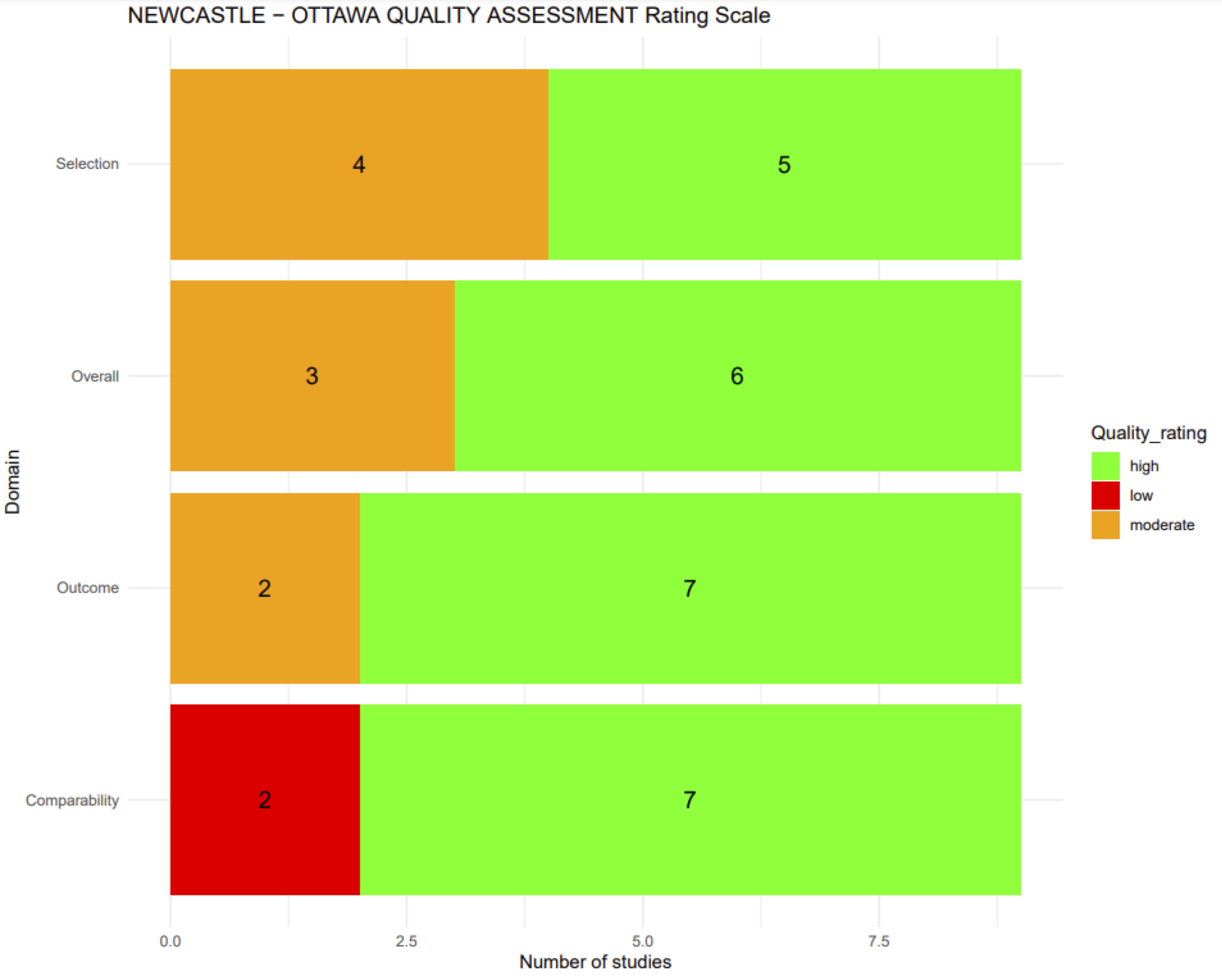

Supplement: S1 Fig — (TIFF) [file pone.0345462.s004.tiff]

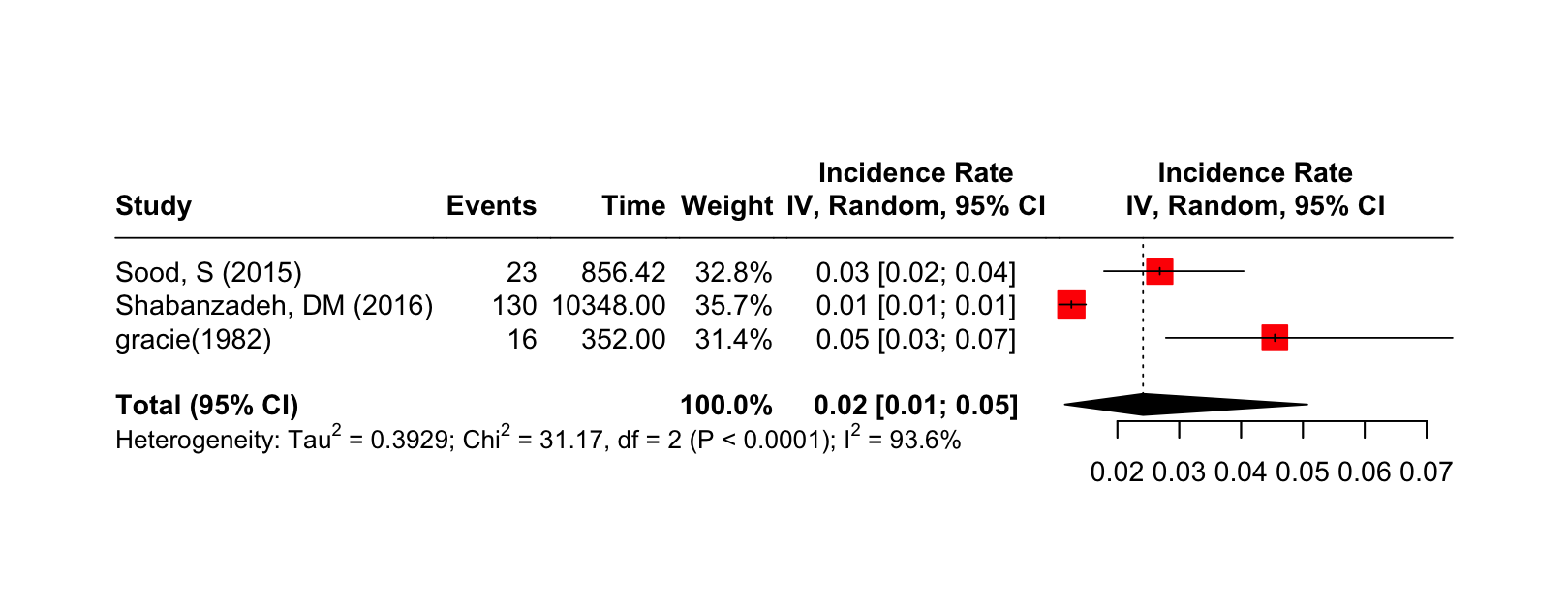

Supplement: S2 Fig — (TIFF) [file pone.0345462.s005.tiff]

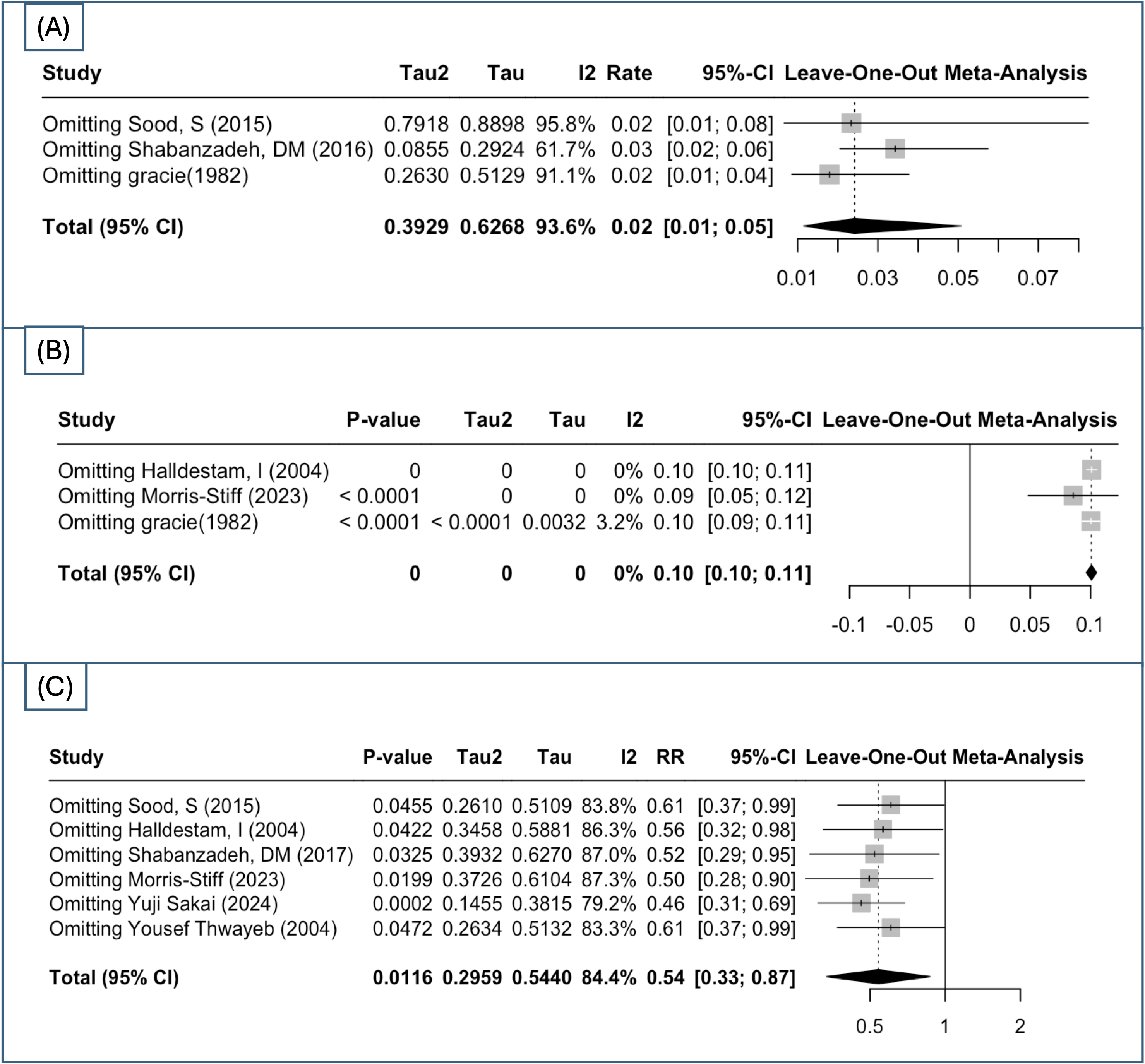

Supplement: S3 Fig — (TIFF) [file pone.0345462.s006.tiff]

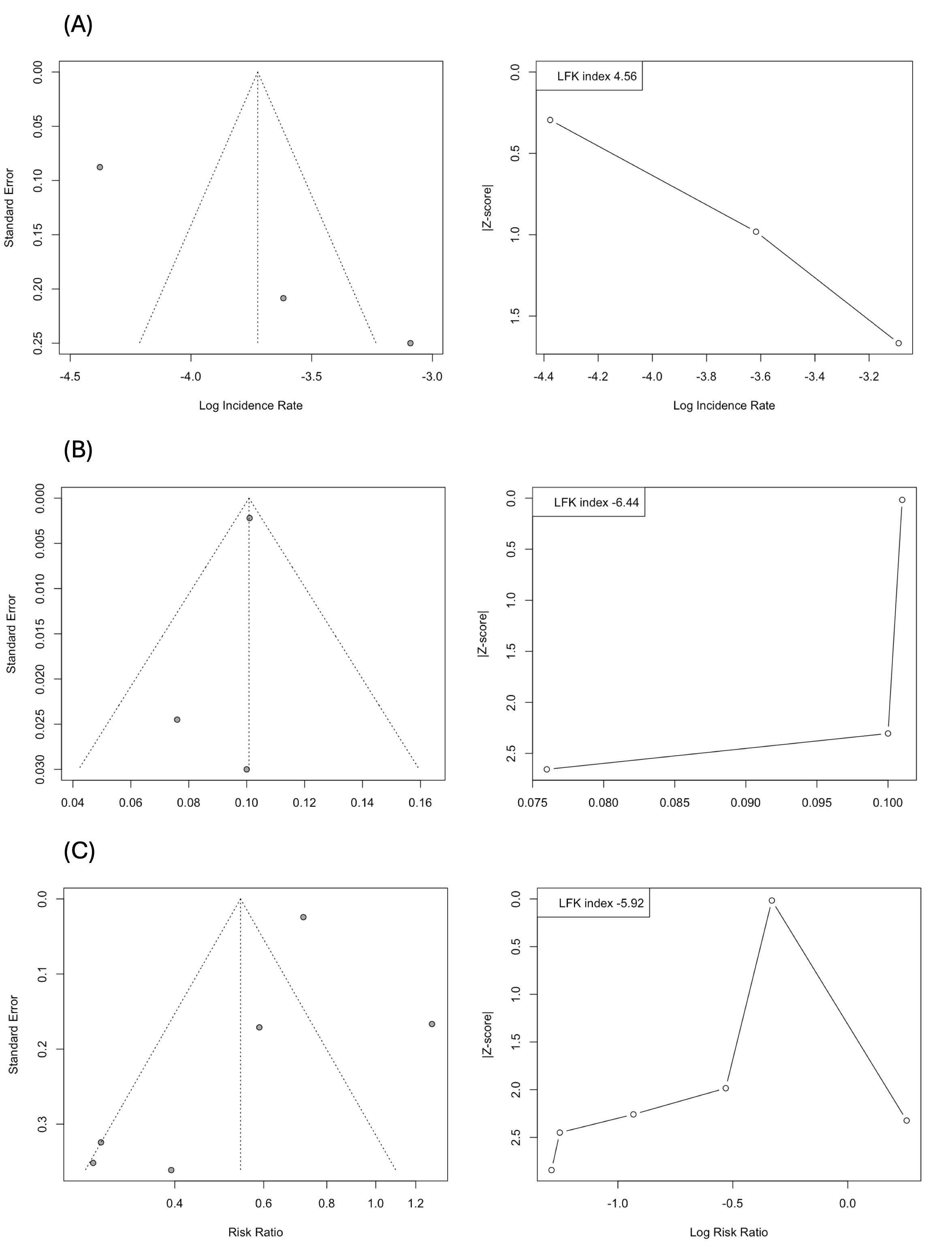

Supplement: S4 Fig — (TIFF) [file pone.0345462.s007.tiff]

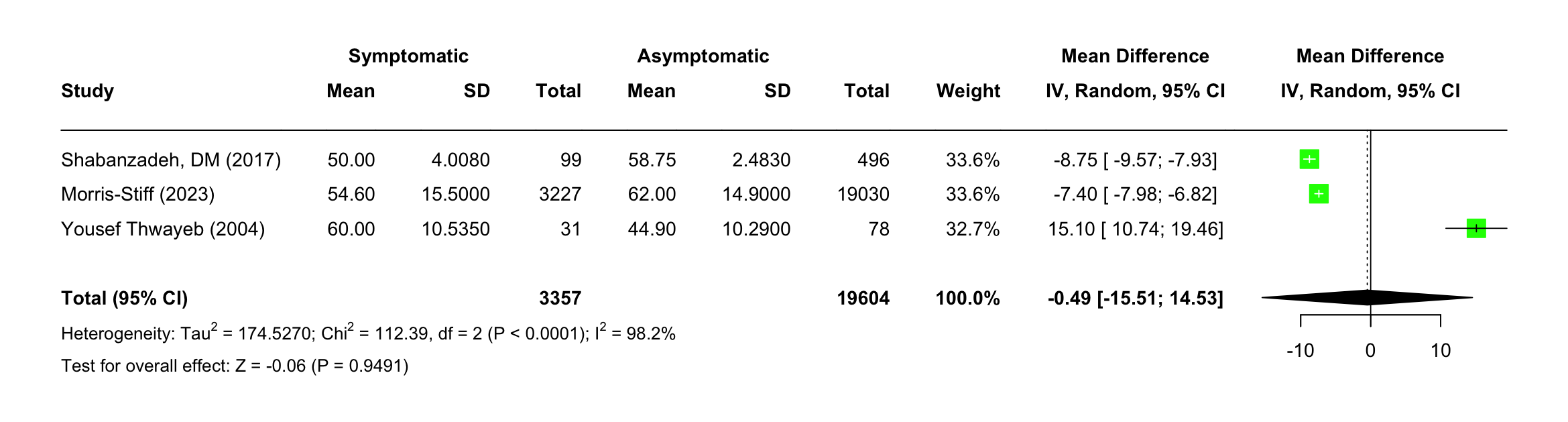

Supplement: S5 Fig — (TIFF) [file pone.0345462.s008.tiff]

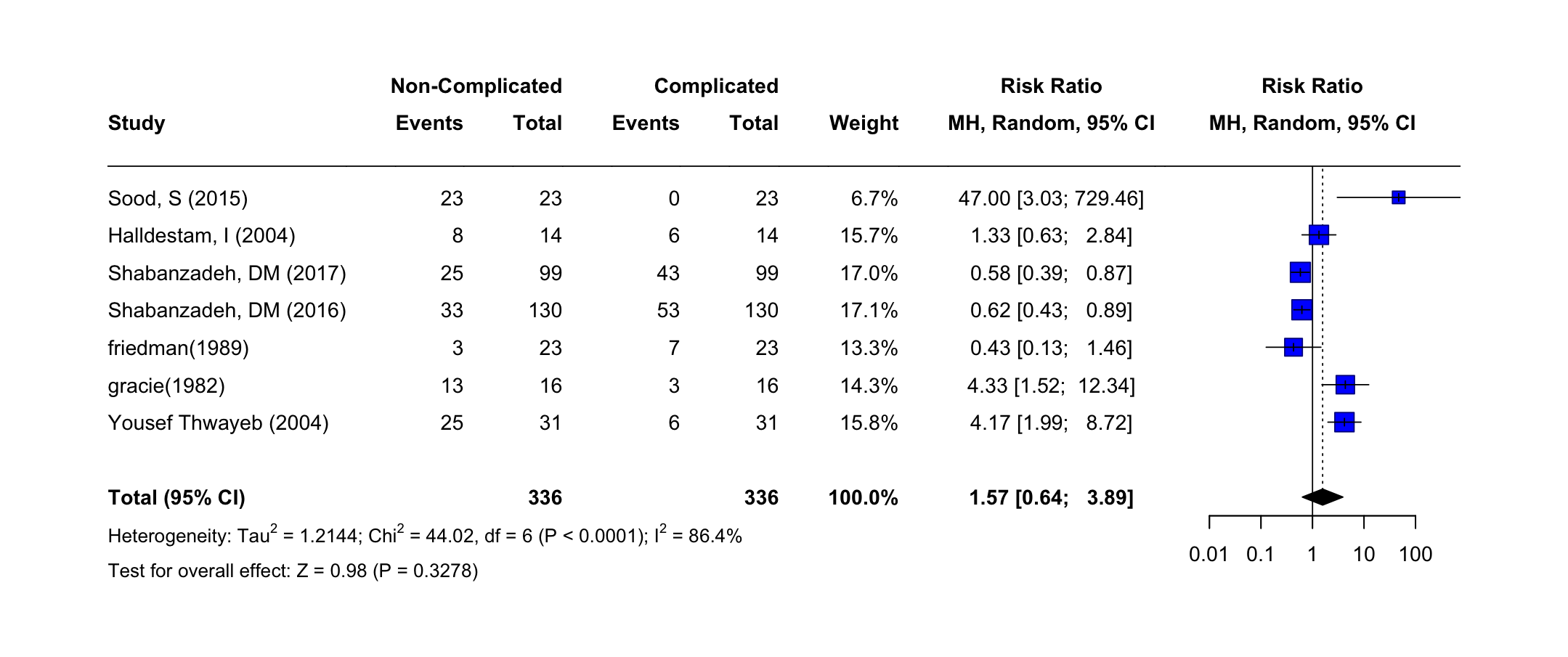

Supplement: S6 Fig — (TIFF) [file pone.0345462.s009.tiff]

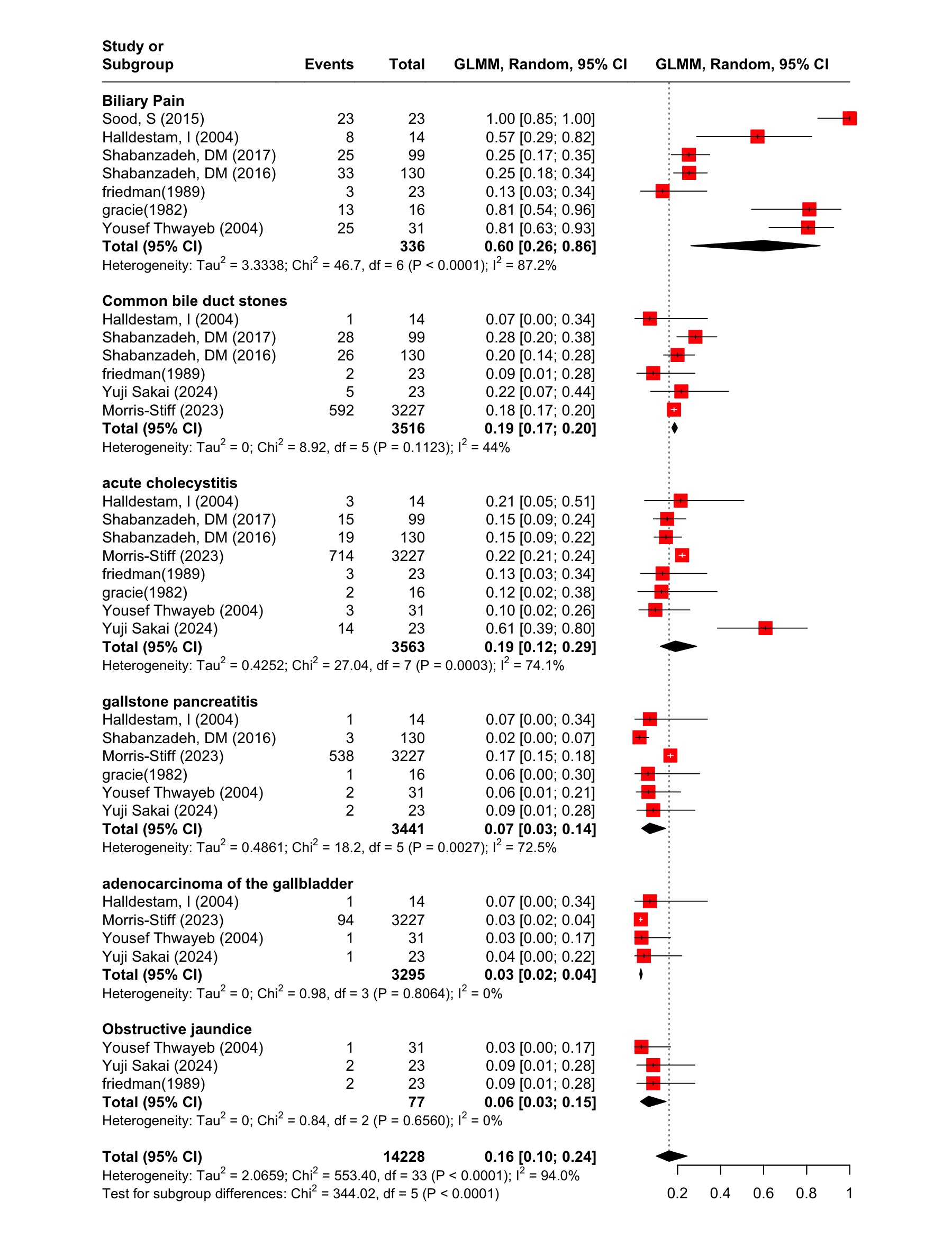

Supplement: S7 Fig — (TIFF) [file pone.0345462.s010.tiff]

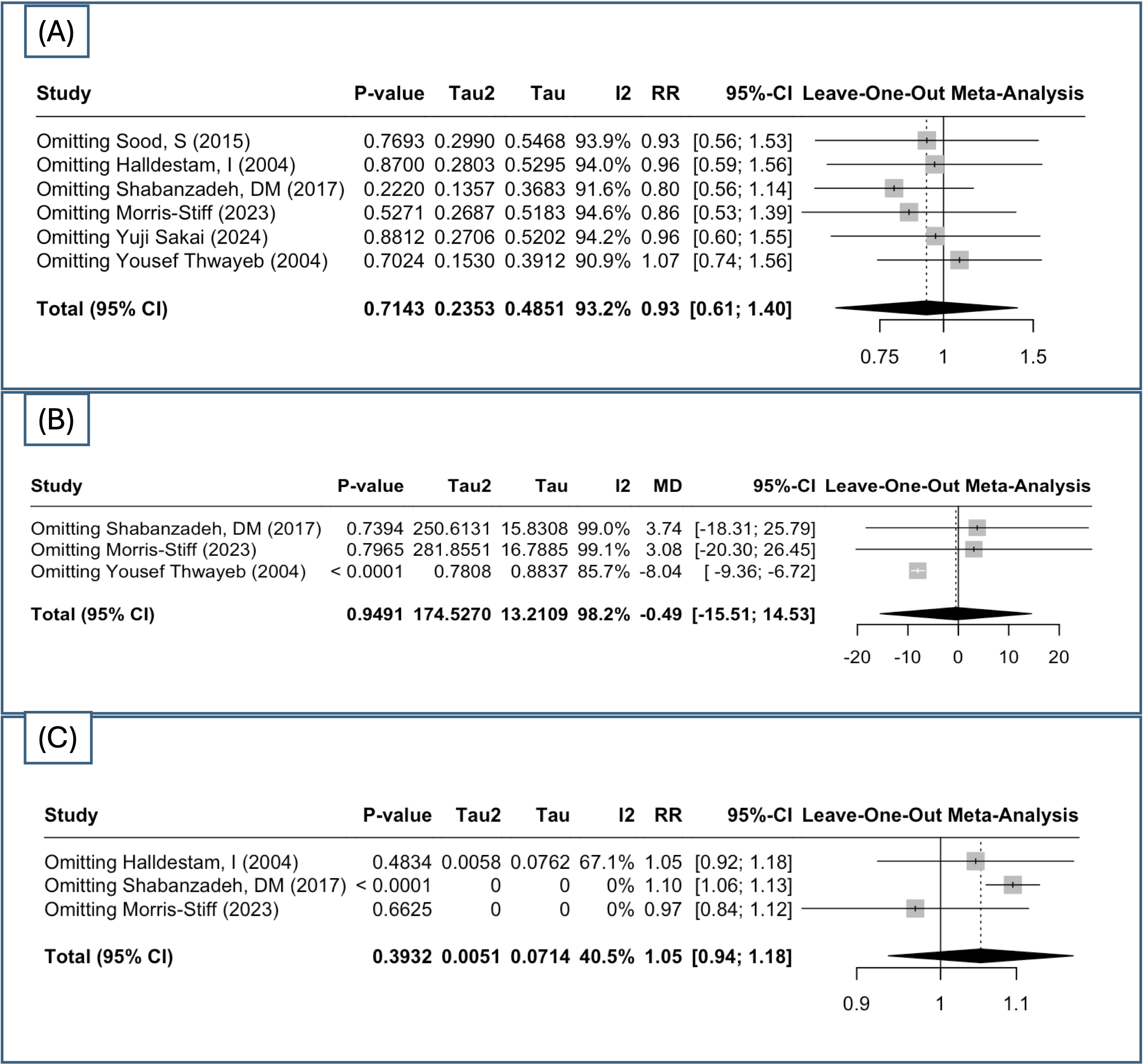

Supplement: S8 Fig — (TIFF) [file pone.0345462.s011.tiff]

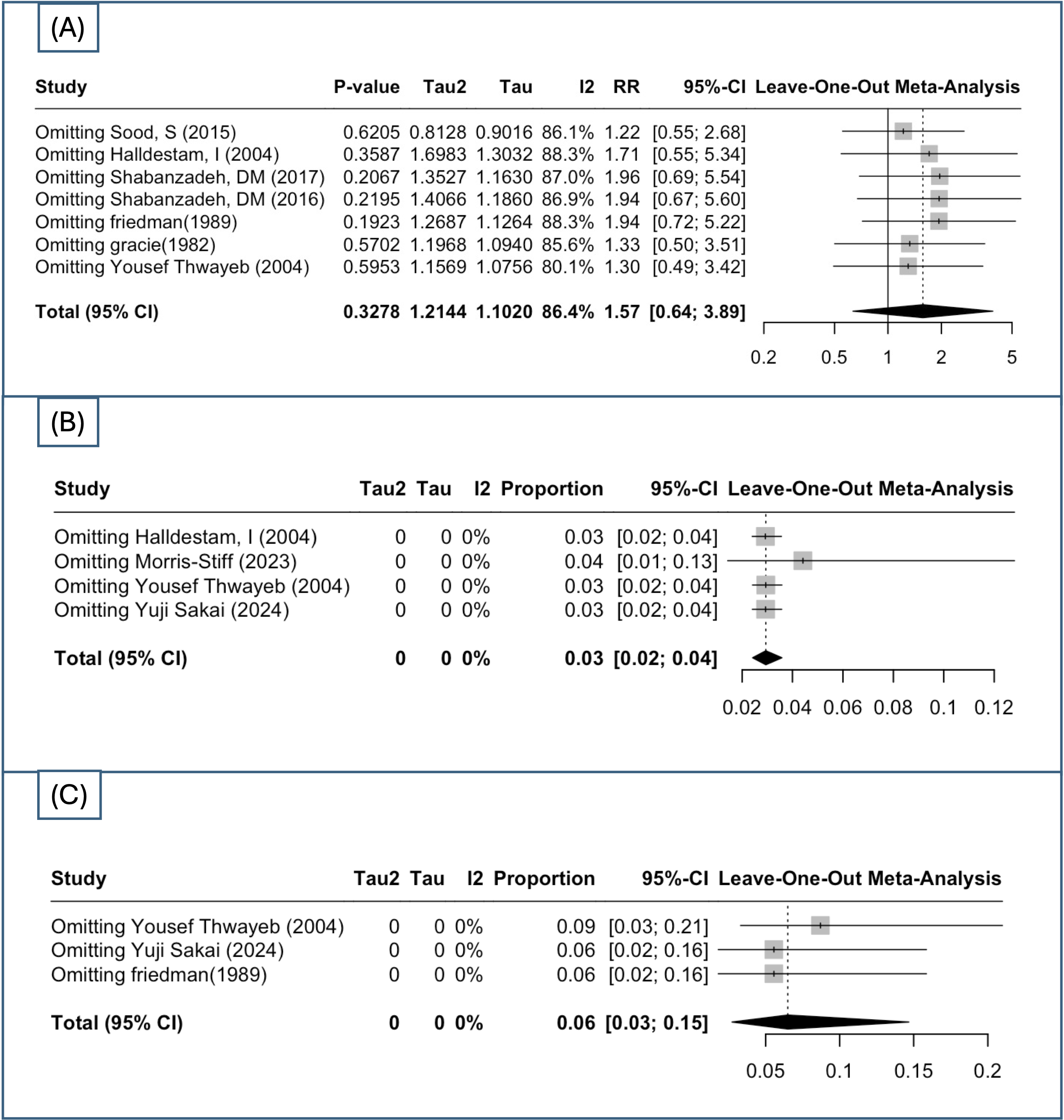

Supplement: S9 Fig — (TIFF) [file pone.0345462.s012.tiff]

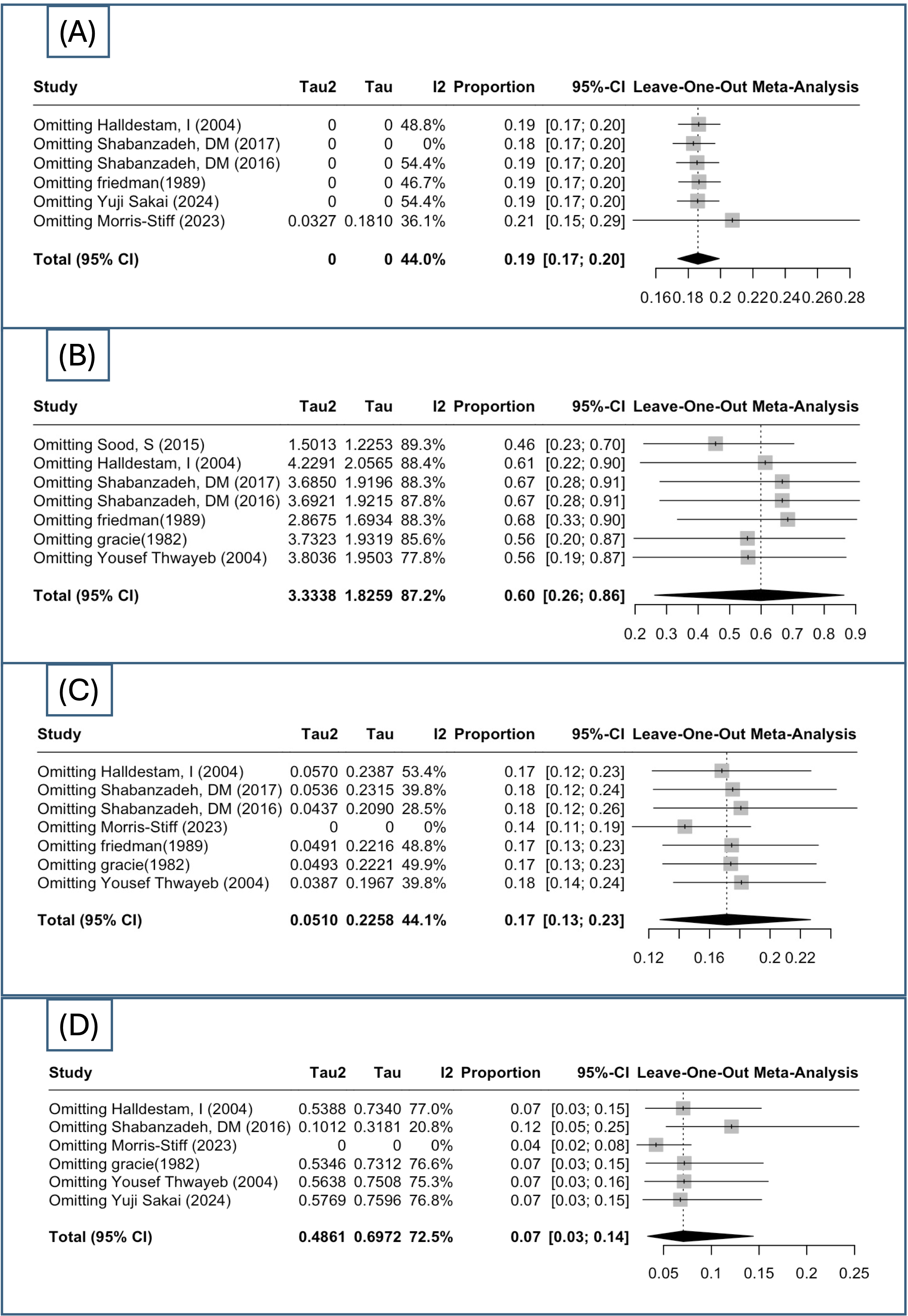

Supplement: S10 Fig — (TIFF) [file pone.0345462.s013.tiff]

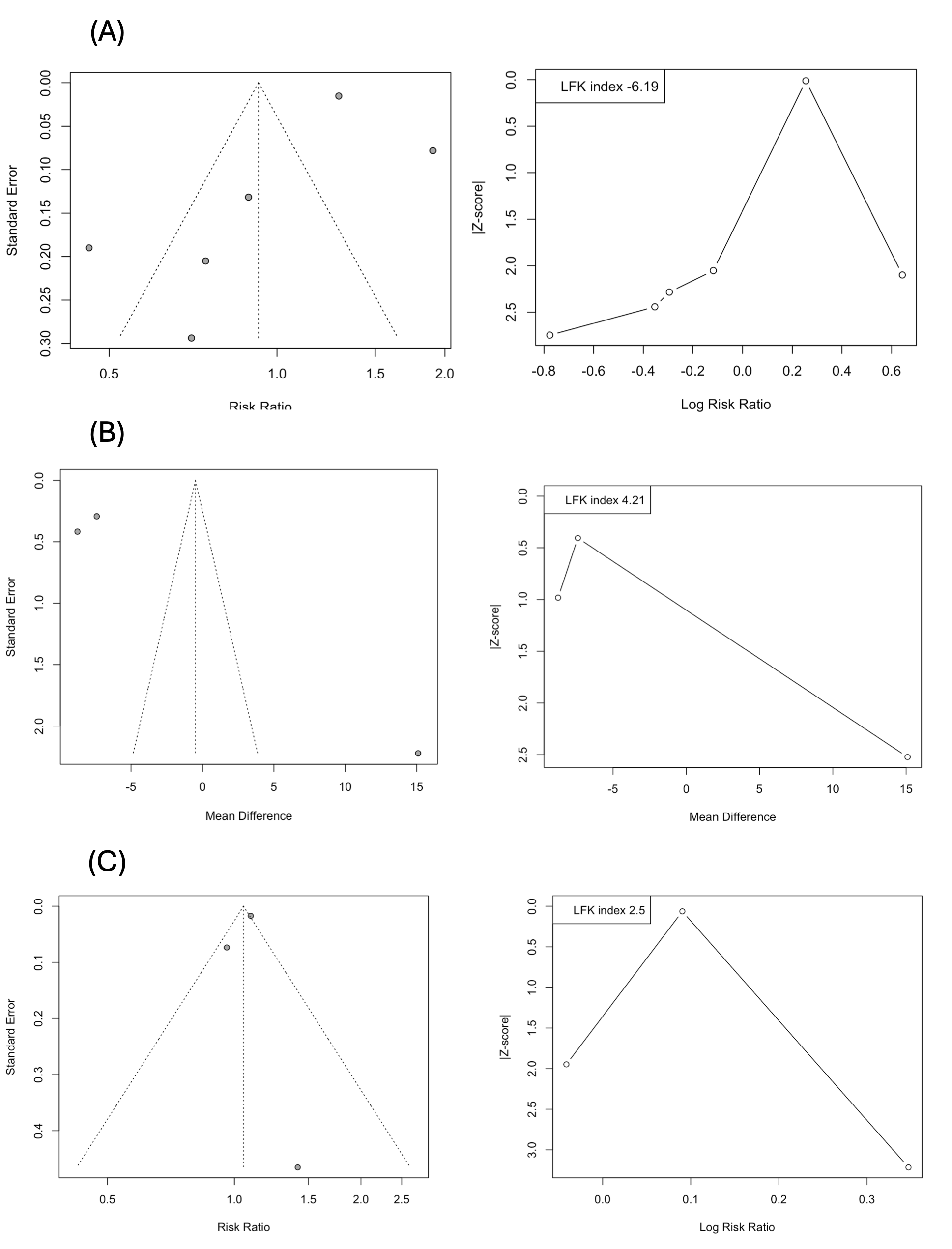

Supplement: S11 Fig — (TIFF) [file pone.0345462.s014.tiff]

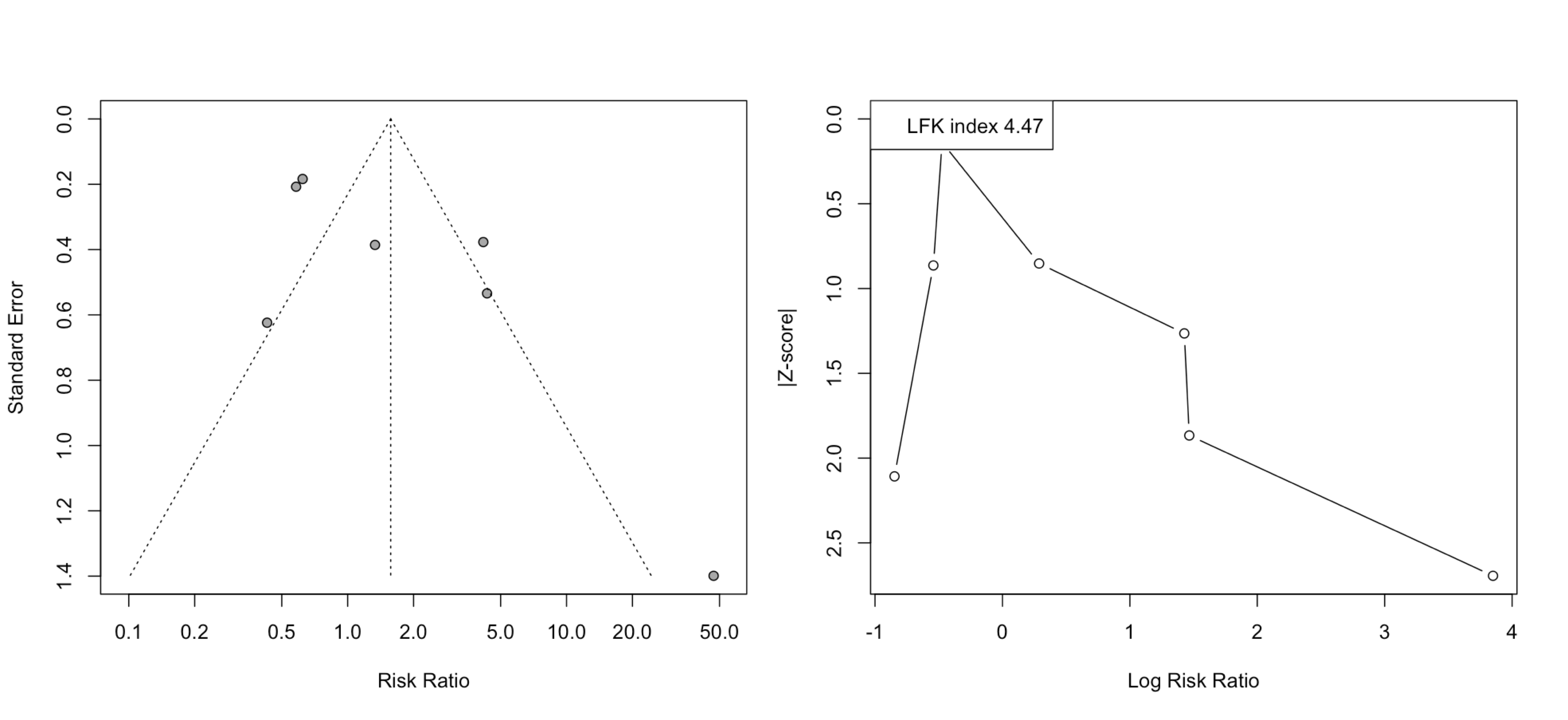

Supplement: S12 Fig — (TIFF) [file pone.0345462.s015.tiff]

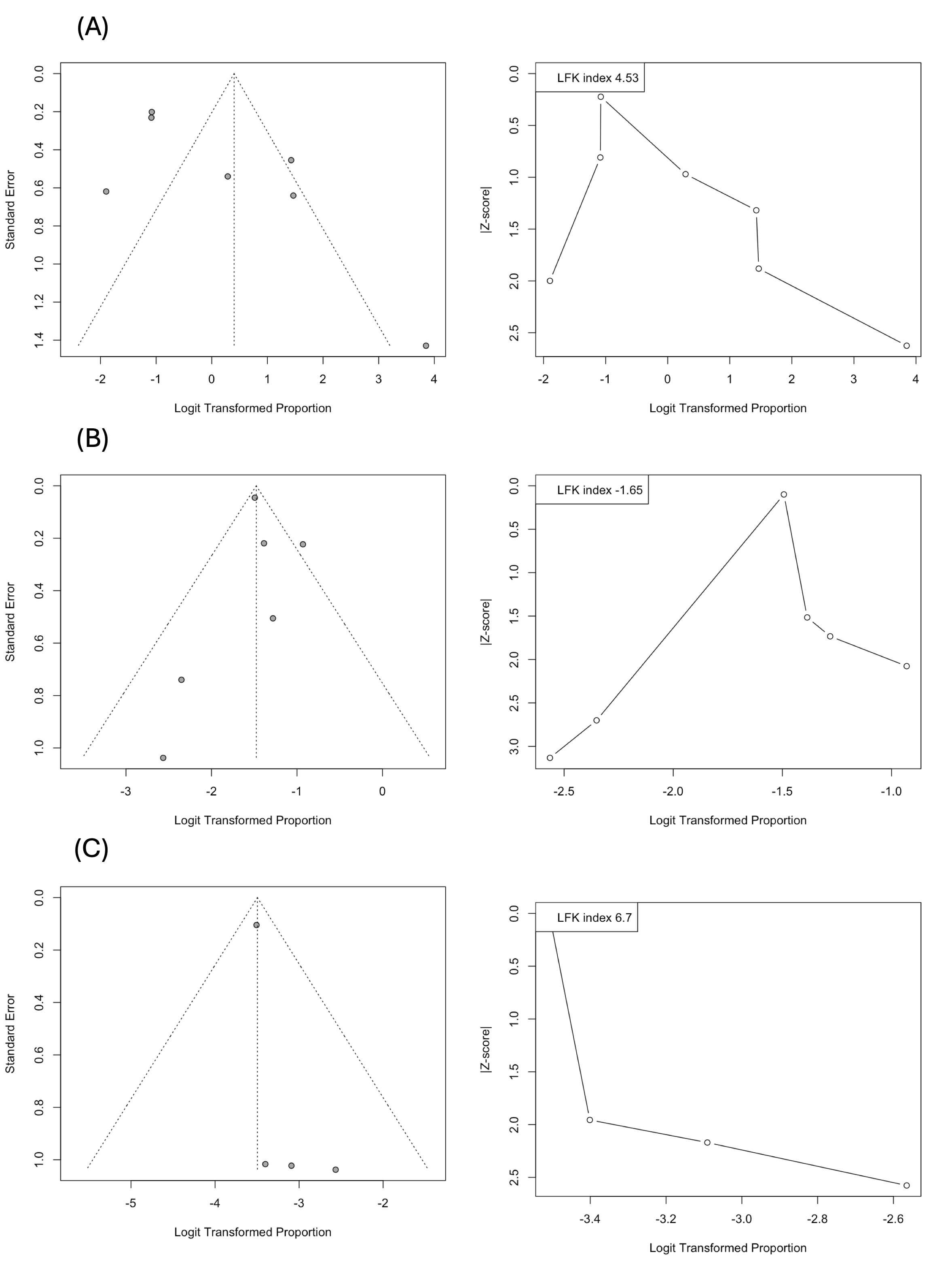

Supplement: S13 Fig — (TIFF) [file pone.0345462.s016.tiff]

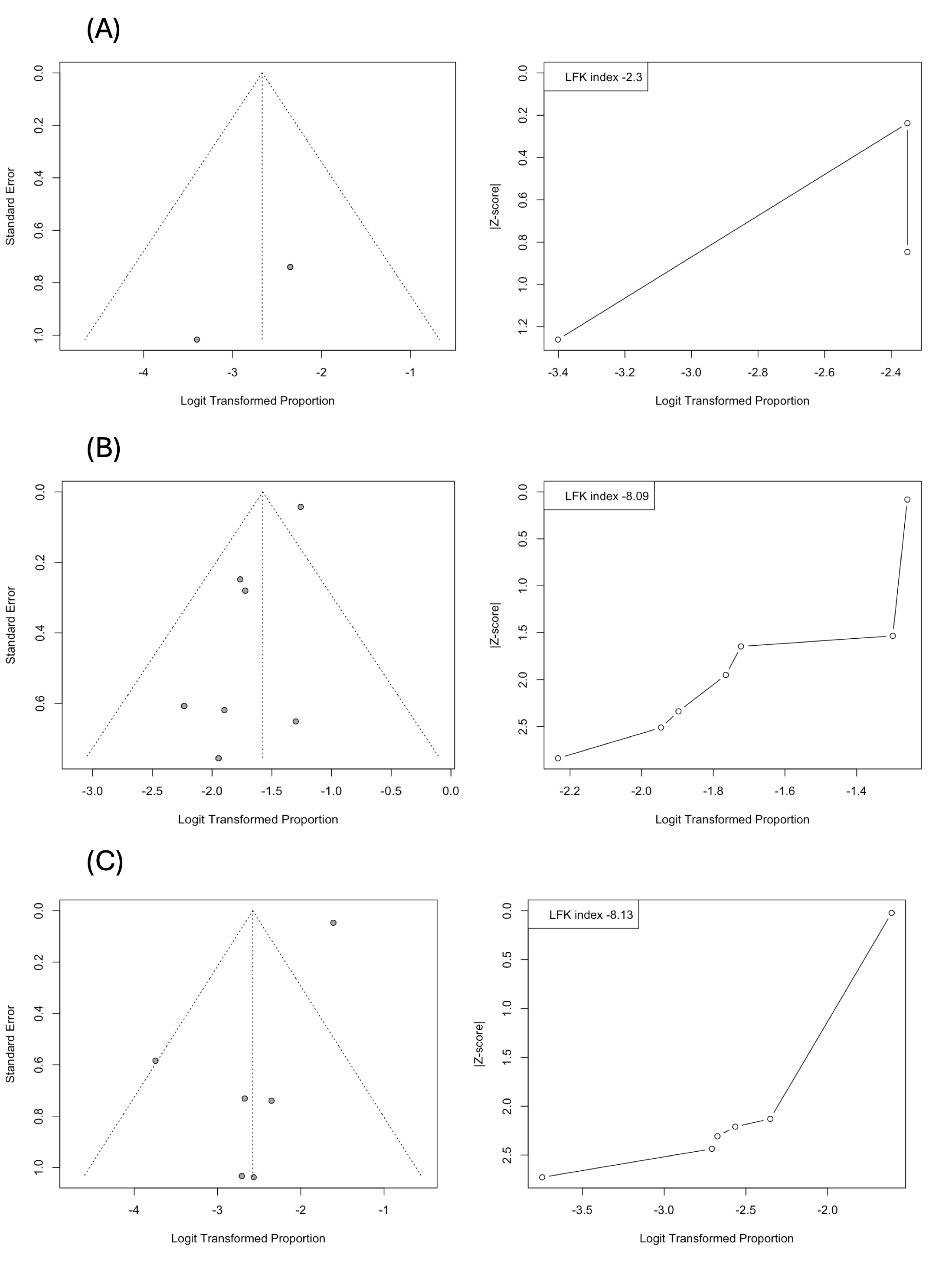

Supplement: S14 Fig — (TIFF) [file pone.0345462.s017.tiff]
