# Supplementary material for: Asymptomatic gallstones: Cumulative incidence proportion, incidence rate, and risk factors for symptoms development: Systematic review and meta-analysis
Source: PLoS One. 2026 Mar 26;21(3):e0345462. doi: 10.1371/journal.pone.0345462 (PMC13020817; doi:10.1371/journal.pone.0345462)
Supplement: Attachment — Submitted filename: Response to Reviewers 2.docx [file pone.0345462.s020.docx]

**Response to Reviewers**

Dear Editorial Team,

We sincerely thank you and the reviewers for your feedback on our manuscript entitled “Asymptomatic Gallstones: Cumulative Incidence Proportion, Incidence Rate, and Risk Factors for Symptoms Development: Systematic Review and Meta-Analysis”, (Manuscript number: PONE-D-25-54208). We have carefully considered all your suggestions and revised the study accordingly. Below, we provide a point-by-point response to each comment.

Point by point responses:

**ACADEMIC EDITOR:**

- The inclusion and exclusion criteria should be meticulously re-evaluated, and the potential limitations arising from these criteria must be discussed. It is crucial to emphasize that factors ranging from the geographical distribution of the included studies to the specific diagnostic modalities employed may significantly impact the interpretation of the analysis. Addressing these points within the Discussion section will substantially enhance the overall quality and clinical relevance of the manuscript.
- **Author’s response:** We thank the academic editor for the insightful points raised, we re-evaluated the inclusion and exclusion criteria per the reviews comments, and refined it accordingly.
- We thank the editor again for the insightful comments regarding the imapct of the geographical variation and diagnostic methods, we furtherly elaborated on these issues and the limitaions araising from each in the discussion section.

**Reviewer’s Comment**

Reviewer #1:

- Have the authors made all data underlying the findings in their manuscript fully available?

Reviewer # 1: No

- **Author’s Response:** During the submission process, we declared that all data used to generate this paper will be available per request, and we are happy to attached our data extraction sheet to the supporting files for the reviewer’s perusal to insure transparency.
- My main problem is still the selection of the articles for the review - or more precisely the assessment of these articles. As I mentioned in my first review the studies are very different ranging from screening of general populations to surgical case-series. Whereas it is quite straight forward to identify asymptomatic gallstones in screened population I still have my doubt about the clinical series. They maybe can be used as support for findings in the population based studies, but not regarded equal to them. In their answer the authors have a long explanation why they find the clinical series to fullfill the criteria for dealing with asymptomatic gallstones, but these arguments should be described in the manuscript. It is of course elegant to use "leave-one-out" approach, but this does not help as regard the quality of the various studies.
- **Author’s Response:** We thank the reviewer for this insightful comment, we added the argument regarding considering these studies sufficient in the discussion section and discussed the limitations arising from the differences between studies ((It is evident that the articles included in this study have different study designs ….. that no ambiguous study was involved in the analysis)), and ((Heterogeneity is also explained by timely influenced evolution …… research nationwide databases)).
- Thank you for provinding me with the Spanish study, but please fill in the correct reference in the Reference-section.
- **Author’s Response:** It has been added, reference no. 32.
- I think I pointed out that it seems as if the Danish study was blinded. I find no comments on this in the revised manuscript, which I find a pity, as this makes the Danish study very important as regard the natural history of asymptomatic gallstones. Given the knowledge of having gallstones could bias people in the interpretation of the reason for abdominal symptoms and thereby change the status of asymptomatic to symptomatic gallstones. As you know there is no clearcut definition of gallstone symptoms.
- **Author’s Response:** Thank you for drawing attention to this point, indeed it is inevitable that the danish study is of higher quality when compared to the other studies, we have included a part in the discussion explaining why the analysis concluded that the danish study was a source of bias even though it is the highest quality paper, and how is that carefully interpreted in our study, ((However, the danish study Shabanzadeh et al.2017 ……strongly emphasizes the need for further studies)).
- Use of oral contraceptive pills is mentioned as an exclusion criteria. Many women used AC pills during these study periods and maybe it is not mentioned in the manuscript. Suggest you leave out this criteria
- **Author’s Response:** We appreciate the reviewer’s insightful comment. We agree that the use of oral contraceptive pills may have been underreported in the included studies and could have introduced unnecessary exclusion. Therefore, this criterion has been removed from the exclusion criteria in the revised manuscript. The eligibility section has been updated accordingly.
- Incidence rate 0.02 - I miss a timeframe.
- **Author’s Response:** Thank you for this observation. We have revised the manuscript to specify that the incidence rate is reported per person-year in the main text to ensure clarity and appropriate interpretation.
- Why do the authers both write 95 % conf. int. and a p-value? conf. int. should be sufficient
- **Author’s Response:** Thank you for this comment. We agree that reporting 95% confidence intervals is sufficient to convey the precision and statistical significance of the results. Therefore, p-values have been removed from the text in the revised manuscript.
- Male gender is protective, but female gender is not a risk factor? Difficult to understand. Please explain.
- **Author’s Response:** Thank you for raising this point, as it is indeed a very critical point that needs to be fully explained to avoid confusion, we added detailed explanations for this issue in the discussion section, (( However, it is important to emphasize on that this study is investigating the development of symptoms in an individual with existent asymptomatic gallstones……by affecting the etiological mechanisms behind gallstones)), and ((there is no scientific evidence proven on why male gender may be a protective factor……. relative to the male population percentages that were reported in the other included studies)).

Reviewer #2:

- Thank you for your hardwork and thorough revision. I appreciate the way you addressed all of the previous comments. The manuscript has improved significantly in clarity, structure, and completeness. I have no further major concerns and believe the paper is now suitable for publication pending the journal’s final editorial checks.
- **Author’s Response:** We would like to thank the reviewer for their input into improving the quality and comprehensiveness of our study.
